# External validation of the KLIC score and development of a novel prediction model for debridement, antibiotics, and implant retention failure in prosthetic joint infection: a multicentre cohort study

**DOI:** 10.5194/jbji-11-591-2026

**Published:** 2026-10-02

**Authors:** Giulia Zumbo, Iris Martínez-Alemany, Jose Maria Bravo-Ferrer, Isabel Nieto Diaz De Los Bernardos, Julia Praena, Alfonso Del Arco, Enrique Nuño, Juan Enrique Corzo Delgado, Francisco Brun, Clara Natera, Alberto Romero Palacios, Beatriz Sobrino, Jesús Rodríguez-Baño, María-Dolores del-Toro-López

**Affiliations:** 1 Unidad Clínica de Enfermedades Infecciosas y Microbiología, Hospital Universitario Virgen Macarena, Departamento de Medicina, Universidad de Sevilla, Instituto de Biomedicina de Sevilla (IBiS)/CSIC, Seville, Spain; 2 CIBERINFEC, Instituto de Salud Carlos III, Madrid, Spain; 3 Unidad Clínica de Traumatología y Cirugía Ortopédica, Hospital Universitario Virgen Macarena, Seville, Spain; 4 Unidad Clínica de Enfermedades Infecciosas, Hospital Universitario Virgen del Rocío, Seville, Spain; 5 Unidad Clínica de Enfermedades Infecciosas, Hospital Costa del Sol, Marbella, Malaga, Spain; 6 Unidad Clínica de Enfermedades Infecciosas, Hospital Virgen de la Victoria, Malaga, Spain; 7 Unidad Clínica de Enfermedades Infecciosas, Hospital de Valme, Sevilla, Spain; 8 Servicio de Medicina Interna, Hospital Universitario Puerta del Mar, Cadiz, Spain; 9 Unidad Clínica de Enfermedades Infecciosas, Hospital Reina Sofía, Cordoba, Spain; 10 Unidad Clínica de Enfermedades Infecciosas, Hospital de Puerto Real, Puerto Real, Cadiz, Spain; 11 Servicio de Enfermedades Infecciosas, Hospital Regional de Málaga, Malaga, Spain

## Abstract

Appropriate estimation of the risk of failure after debridement, antibiotics, and implant retention (DAIR) may provide useful prognostic information in patients with prosthetic joint infection (PJI). We aimed to externally validate the Kidney, Liver, Index surgery, Cemented prosthesis, C-reactive protein (KLIC) score and to develop and internally validate a new prediction model based on variables available before DAIR. We conducted a retrospective multicentre cohort study using medical record data from adults with PJI treated with DAIR across eight Spanish centres between 2006 and 2023, with a minimum 12-month follow-up. Predictors were analysed using a generalized linear mixed-effects model, with assessment of discrimination, calibration, and bootstrap internal validation. Among 224 patients, 121 (54 %) experienced DAIR failure. The final model retained revision prosthesis, Charlson Comorbidity Index ≥ 3, C-reactive protein > 150 mg L^−1^, longer time from index arthroplasty to DAIR, and Gram-negative bacilli or polymicrobial infection. The model showed good discrimination (Areas under the receiver operating characteristic curve (AUC-ROC) 0.85; 95 % confidence interval (CI) 0.79–0.90) and satisfactory calibration; the optimism-corrected AUC-ROC was 0.83. The derived clinical score showed an AUC-ROC of 0.81 (95 % CI 0.75–0.87) and identified groups with observed failure rates of 25.5 %, 45.6 %, and 87.9 %. The KLIC score did not demonstrate discriminative ability beyond chance (AUC-ROC 0.53; 95 % CI 0.44–0.64). Excluding microbiological aetiology reduced discrimination to 0.81 for the multivariable model and 0.77 for the corresponding reduced score. The new model showed good discrimination and calibration in this cohort, but external validation in independent PJI-DAIR populations is required before clinical implementation can be recommended.

## 1 Introduction

Prosthetic joint infection (PJI) remains one of the most serious complications following arthroplasty, with a substantial impact on patient morbidity, functional outcomes, quality of life, and healthcare costs (Patel, 2023). The incidence of PJI within the first 2 years after primary arthroplasty ranges from 0.5 % to 2.3 % (Parel et al., 2025; MAC, 2020), increasing to 5.5 %–8.1 % and 6.2 %–8.4 % following revision total hip and total knee arthroplasty, respectively (Lenguerrand et al., 2019; Quinlan et al., 2020).

Debridement, antibiotics, and implant retention (DAIR) is an established surgical strategy for selected patients with acute PJI, allowing preservation of the prosthesis. Improvements in surgical technique, including exchange of modular components, together with optimized antimicrobial therapy, have contributed to improved outcomes (Löwik et al., 2020; Rahardja et al., 2023). Nevertheless, reported failure rates remain highly variable, ranging from 7 % to 55 % (Sigmund et al., 2025), reflecting differences in patient characteristics, infection-related factors, treatment strategies, outcome definitions, and duration of follow-up (Abbaszadeh et al., 2025; Kristensen et al., 2025; Löwik et al., 2020; Rahardja et al., 2023).

Accurate estimation of the risk of DAIR failure before surgery could therefore provide useful prognostic information when treatment strategies are being considered. In 2015, Tornero et al. developed the KLIC score (Kidney, Liver, Index surgery, Cemented prosthesis, C-reactive protein), a pragmatic prediction tool based on readily available preoperative variables (Tornero et al., 2015). Subsequent external validation studies, however, have shown inconsistent discrimination, with reported areas under the receiver operating characteristic curve (AUC-ROC) ranging from 0.53 to 0.76 (Bernaus et al., 2022; Jiménez-Garrido et al., 2018; Liukkonen et al., 2024; Löwik et al., 2018). A previous multicentre study involving our group identified predictors of treatment failure among patients with *Staphylococcus aureus* PJI, including a DAIR-specific multivariable model with good discrimination. However, its applicability was restricted to *S. aureus* infections, and the model incorporated treatment-related variables not necessarily available before DAIR (Espíndola et al., 2022).

We therefore aimed to externally validate the KLIC score in a multicentre cohort of patients with PJI treated with DAIR and to develop and internally validate a new prediction model based on variables available before DAIR.

## 2 Methods

### 2.1 Study design and population

We conducted a retrospective, multicentre cohort study including adult patients ( ≥18 years) diagnosed with PJI according to internationally accepted criteria (McNally et al., 2021) and treated with DAIR as the initial surgical strategy across eight participating Spanish centres between January 2006 and December 2023. This study was reported in accordance with the TRIPOD + AI 2024 reporting guideline (Collins et al., 2024), and the completed checklist is provided as Table S1 in the Supplement.

The study was approved by the competent Research Ethics Committee (PR-PI0454-2012). A single favourable ethics opinion was valid for all participating centres within the Andalusian Public Health System. The approval covered retrospective data collection under the study protocol, which continued throughout the study period without requiring additional centre-specific approvals or protocol amendments. The requirement for informed consent was waived by the Ethics Committee owing to the retrospective nature of the study.

### 2.2 Data collection

Demographic characteristics, comorbidities, laboratory parameters, surgical variables, and microbiological data available before DAIR were retrospectively collected from medical records using a previously established PJI database with standardized definitions. Comorbidity burden was assessed using the Charlson Comorbidity Index (CCI). Time to DAIR was defined as the interval, in days, from the index arthroplasty to the DAIR procedure.

### 2.3 Outcome definition and follow-up

The primary outcome was DAIR failure, defined as the occurrence of at least one of the following during follow-up: (1) persistence or recurrence of signs or symptoms of infection, (2) requirement for further revision surgery (implant exchange or removal), (3) need for long-term suppressive antimicrobial therapy, or (4) infection-related mortality.

Patients were followed for a minimum of 12 months after DAIR or until confirmed treatment failure.

### 2.4 Statistical analysis

Continuous variables were summarized as mean with standard deviation (SD) or medians with interquartile ranges (IQRs), as appropriate, and categorical variables as counts and percentages. Comparisons between groups were performed using the Student's t test or Mann–Whitney U test for continuous variables and the $\chi^{2}$ test or Fisher's exact test for categorical variables, as appropriate. Continuous predictors were initially evaluated in their original form and, where appropriate, alternative parameterizations, including clinically relevant categorizations, were explored. CCI was initially evaluated continuously; alternative cut-points were subsequently assessed according to the distribution of failure, strength of association, and model discrimination, with CCI ≥ 3 providing the best predictive performance. Alternative C-reactive protein (CRP) cut-points were similarly explored; >150 mg L^−1^, a threshold previously associated with DAIR failure (Wouthuyzen-Bakker et al., 2019), provided the best predictive contribution and was retained. Time from index arthroplasty to DAIR was retained as a continuous variable.

Variables associated with DAIR failure in univariable analysis ( p<0.10 ), together with variables considered clinically relevant based on prior evidence, were considered for multivariable modelling. A generalized linear mixed-effects model with a logit link and hospital as a random intercept was used to account for clustering by centre. Adjusted odds ratios (ORs) with 95 % confidence intervals (CIs) were reported. The primary model was fitted using complete cases.

Missing data were almost exclusively restricted to CRP (38 patients), with one additional missing CCI value. To assess the robustness of the complete-case analysis, multiple imputation by chained equations was performed ( m=40 ). Continuous CRP values were imputed before applying the 150 mg L^−1^ threshold. The imputation model included the outcome, predictors included in the final model, calendar period, and hospital. The final mixed-effects model was refitted in each imputed dataset, and estimates were pooled using Rubin's rules.

Model discrimination was evaluated using the area under the receiver operating characteristic curve (AUC-ROC). Calibration was assessed using a calibration plot comparing predicted and observed probabilities across risk deciles and the Hosmer–Lemeshow goodness-of-fit test. Internal validation was performed using 1000 bootstrap resamples. In each resample, the mixed-effects model was refitted, and its performance was evaluated in both the bootstrap sample and the original dataset. Mean optimism across resamples was used to obtain optimism-corrected estimates of discrimination and calibration.

A clinical risk score was derived from the fixed-effect regression coefficients ( β ) of the final model. The coefficient for time to DAIR ( β=0.014 d^−1^) was used as the scaling reference. Half of this coefficient (0.007) was assigned 0.01 score units, and each coefficient was transformed as (β/0.007)×0.01 . Categorical weights were rounded to the nearest 0.5 point. To facilitate clinical calculation, all weights were subsequently rescaled by a factor of 2, yielding 3 points for revision prosthesis, 5 points for CCI ≥ 3, 4 points for CRP > 150 mg L^−1^, 4 points for Gram-negative bacilli or polymicrobial infection, and days to DAIR/25 points (1 point per 25 d to DAIR). Thus, the final score was calculated as 3 (revision prosthesis) + 5 (CCI ≥ 3) + 4 (CRP > 150 mg L^−1^) + (Gram-negative / polymicrobial infection) + days to DAIR / 25.

Risk categories were empirically defined from observed failure rates across increasing score values as low risk ( ≤4 ), intermediate risk ( >4 to <8 ), and high risk ( ≥8 ). Because these thresholds were derived in the development cohort, they were considered exploratory.

Several sensitivity analyses were performed. First, calendar period (2006–2014 vs 2015–2023) was added as a fixed effect to the final model to assess potential temporal heterogeneity. Second, the model was refitted with Gram-negative bacilli and polymicrobial infection entered as separate predictors. Third, microbiological aetiology was excluded, and a corresponding reduced clinical score was derived using the same weighting procedure. Finally, the multiple-imputation analysis described above was used to assess the impact of missing data.

KLIC scores were calculated according to the original published algorithm. The discriminative performance of the derived clinical scores and the KLIC score was assessed in the same cohort using AUC-ROC without hospital-level adjustment, allowing direct comparison of score performance.

Calibration, internal validation by bootstrap resampling, and multiple imputation by chained equations were performed in R (R Foundation for Statistical Computing, Vienna, Austria); all other statistical analyses were performed using SPSS Statistics version 29.0 (IBM Corp., Armonk, NY, USA). A two-sided p<0.05 was considered statistically significant.

### 2.5 Sample size and data use

No formal a priori sample-size calculation was performed; all eligible patients identified during the study period were included. The full development dataset was used for model fitting without random partitioning into training and test sets; internal validation was performed by bootstrap resampling.

## 3 Results

### 3.1 Cohort characteristics and outcome

A total of 224 patients from eight participating centres were included. The median age was 71 years (IQR 64–78), and 91 (40.6 %) were male. The most frequently identified pathogen was *Staphylococcus aureus* ( n=65 , 29 %), followed by coagulase-negative staphylococci ( n=42 , 18.8 %), Gram-negative bacilli ( n=42 , 18.8 %), and polymicrobial infections ( n=25 , 11.2 %). Overall, DAIR failure occurred in 121 patients (54.0 %). Median follow-up from DAIR was 657 d (IQR 381–852), corresponding to 21.6 months (IQR 12.5–28.0). Baseline characteristics are summarized in Table 1.

**Table 1:** Baseline characteristics of patients with postoperative PJI treated with DAIR.

Variable	Total ( N=224 )
Demographic and host-related characteristics	
Male sex	91 (40.6)
Age (years), median (IQR)	71 (64–78)
Charlson Comorbidity Index, median (IQR)	1 (0–1)
Charlson Comorbidity Index ≥ 3, n (%)	23 (10.3)
Laboratory parameters at diagnosis	
Haemoglobin (g dL^−1^), median (IQR)	10.6 (9.5–11.6)
Leucocyte count (cell mm^−3^), median (IQR)	8470 (6500–11 450)
CRP (mg L^−1^), median (IQR)	75.5 (26–150)
CRP > 150 mg L^−1^	46/186 (24.7)
Surgical characteristics	
Fracture as indication of arthroplasty	60 (26.8)
Hip PJI	117 (52.3)
Revision prosthesis	71 (31.7)
Cemented prosthesis	142/190 (74.7)
Days to DAIR, median (IQR)	36 (22–65)
Follow-up from DAIR in days, median (IQR)	657 (381–852)
Microbiological features	
Bacteraemia at PJI diagnosis	17/221 (7.7)
Aetiology	
Negative cultures	22 (9.8)
*Staphylococcus aureus*	65 (29)
Coagulase-negative staphylococci	42 (18.8)
*Streptococcus/Enterococcus* spp.	18 (8)
Gram-negative bacilli	42 (18.8)
Polymicrobial	25 (11.2)
Anaerobes	6 (2.7)
Others	4 (1.8)
Outcome	
DAIR failure	121 (54 %)

Data are presented as number (percentage) unless otherwise indicated. Denominators vary due to missing data and are indicated where applicable. CRP: C-reactive protein; PJI: prosthetic joint infection; DAIR: debridement, antibiotics, and implant retention.

### 3.2 Univariable analysis

In univariable analysis, CCI ≥ 3, CRP > 150 mg L^−1^, revision prosthesis, and longer time from index arthroplasty to DAIR were associated with DAIR failure (Table 2). Gram-negative bacilli and polymicrobial infections showed higher observed failure rates (66.7 % and 68.0 %, respectively), although the individual associations did not reach statistical significance.

**Table 2:** Univariable analysis of factors associated with DAIR failure.

Categorical variables				
	Failure n/N (%)	No failure n/N (%)	OR (95 % CI)	p value
Male sex	52/91 (57.1)	39/91 (42.9)	0.80 (0.5–1.4)	0.438
Charlson Comorbidity Index ≥ 3	18/23 (78.3)	5/23 (21.7)	3.39 (1.2–9.5)	0.015
CRP > 150 mg L^−1^	32/46 (69.6)	14/46 (30.4)	2.29 (1.1–4.6)	0.021
Bacteraemia	12/17 (70.6)	5/17 (29.4)	2.13 (0.7–6.3)	0.161
Fracture indication	31/60 (51.7)	29/60 (48.3)	0.88 (0.5–1.6)	0.669
Revision prosthesis	51/71 (71.8)	20/71 (28.2)	3.02 (1.6–5.5)	<0.001
Cemented prosthesis	75/142 (52.8)	67/142 (47.2)	0.67 (0.3–1.3)	0.243
Hip PJI	67/117 (57.3)	50/117 (42.7)	1.34 (0.8–2.3)	0.277
Gram-negative infection	28/42 (66.7)	14/42 (33.3)	1.90 (0.9–3.9)	0.068
Polymicrobial infection	17/25 (68.0)	8/25 (32.0)	1.90 (0.8–4.7)	0.137
Continuous variables				
	Failure median (IQR)	No failure median (IQR)	p value	
Age (years)	70 (63–79)	70 (65–77)	0.545	
Charlson Comorbidity Index	1 (0–1)	1 (0–1)	0.098	
Haemoglobin (g dL^−1^)	10.5 (9.4–11.6)	10.6 (9.5–11.6)	0.457	
Leucocyte count (cell mm^−3^)	8250 (6420–11 570)	8780 (6503–12 615)	0.920	
CRP (mg L^−1^)	77.5 (27–187)	61.5 (23–133)	0.023	
Time from index arthroplasty to DAIR (days)	43 (27–208)	31 (20–39)	<0.001	

Denominators vary due to missing data and are shown where applicable. CRP: C-reactive protein; DAIR: debridement, antibiotics, and implant retention.

### 3.3 Primary multivariable model

The primary multivariable analysis included 185 patients (82.6 %) with complete data and 102 failure events. After accounting for hospital-level clustering, the final model retained revision prosthesis (OR 2.64; 95 % CI 1.20–5.81; p=0.017 ), CCI ≥ 3 (19/185 patients; OR 5.15; 95 % CI 1.25–21.22; p=0.024 ), CRP > 150 mg L^−1^ (OR 4.81; 95 % CI 1.98–11.67; p<0.001 ), longer time from index arthroplasty to DAIR (per day increase; OR 1.01; 95 % CI 1.01–1.02; p=0.001 ), and Gram-negative bacilli or polymicrobial infection (OR 4.03; 95 % CI 1.77–9.19; p=0.001 ) (Table 3).

**Table 3:** Multivariable analysis of factors associated with DAIR failure and derived clinical score.

Variable	β	Odds	95 % CI	p value	Score
	coefficient	ratio			
Revision prosthesis	0.970	2.64	1.20–5.81	0.017	3
Charlson Comorbidity Index ≥ 3	1.638	5.15	1.25–21.22	0.024	5
CRP > 150 mg L^−1^	1.570	4.81	1.98–11.67	<0.001	4
Time from index arthroplasty to DAIR, d^−1^	0.014	1.01	1.01–1.02	0.001	days/25 (0.04 d^−1^)
Gram-negative bacilli or polymicrobial infection	1.393	4.03	1.77–9.19	0.001	4

CCI ≥ 3 was present in 19/185 (10.3 %) patients included in the complete-case analysis (23/224 (10.3 %)) in the overall cohort). The final model included five predictors and 102 failure events. CRP: C-reactive protein; DAIR: debridement, antibiotics, and implant retention.

### 3.4 Model performance and internal validation

The final mixed-effects model showed good discrimination, with an AUC-ROC of 0.85 (95 % CI 0.79–0.90) (Fig. 1A). Visual assessment of the calibration plot showed satisfactory agreement between predicted and observed probabilities (Fig. 1B), and the Hosmer–Lemeshow test showed no evidence of lack of fit ( p=0.53 ). Bootstrap internal validation showed limited optimism, with an optimism-corrected AUC-ROC of 0.83 compared with an apparent AUC-ROC of 0.85, and an optimism-corrected calibration slope of 0.91.

**Figure 1:**
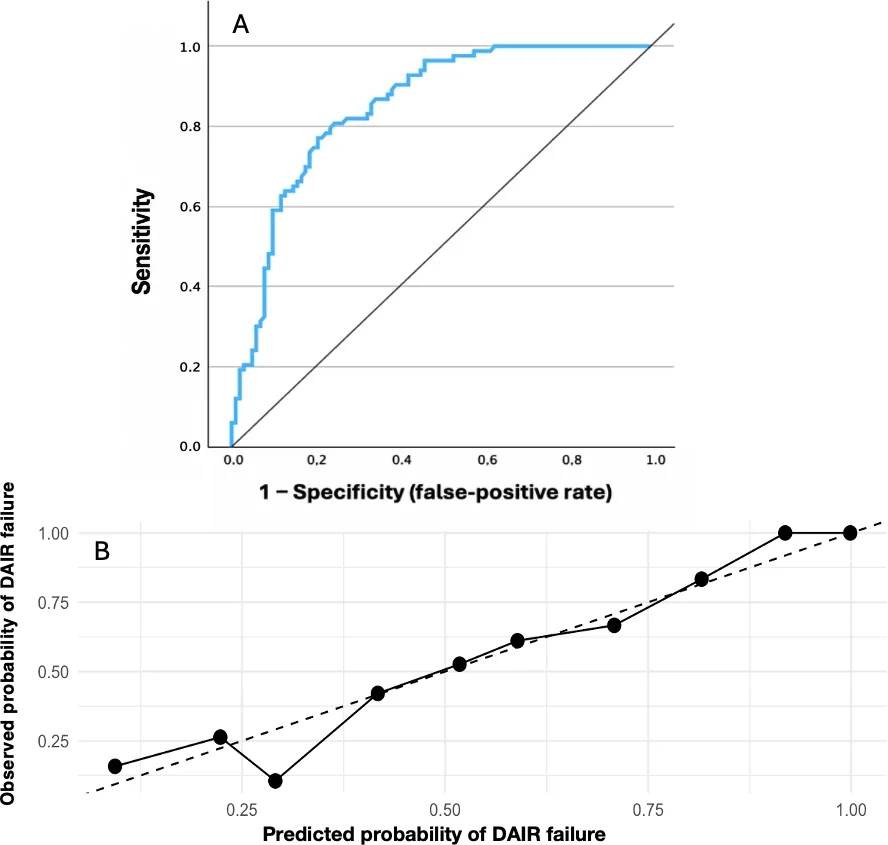
Performance of the multivariable prediction model. **(A)** Receiver operating characteristic (ROC) curve showing model discrimination (AUC-ROC 0.85; 95 % CI 0.79–0.90). **(B)** Calibration plot comparing predicted and observed probabilities of DAIR failure across deciles of predicted risk.

### 3.5 Derived clinical score and KLIC

The derived clinical score yielded an AUC-ROC of 0.81 (95 % CI 0.75–0.87). In contrast, the KLIC score did not demonstrate discriminative ability beyond chance in this cohort (AUC-ROC 0.53; 95 % CI 0.44–0.64) (Fig. 2).

**Figure 2:**
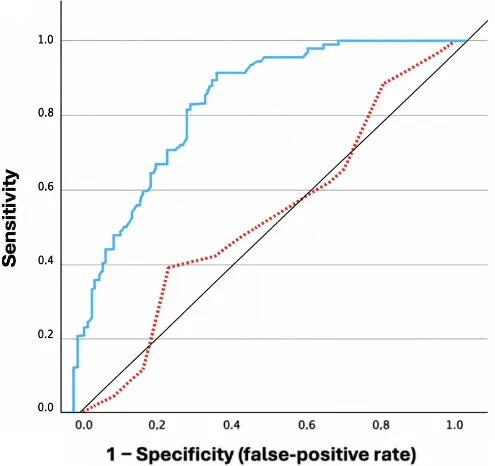
Discriminative performance of the derived clinical score and KLIC score. Receiver operating characteristic (ROC) curves showing an AUC of 0.81 (95 % CI 0.75–0.87) for the derived clinical score (solid line) and 0.53 (95 % CI 0.44–0.64) for the KLIC score (dashed line).

### 3.6 Risk stratification

Using the empirically derived thresholds, observed failure rates were 25.5 % (13/51; 95 % CI 15.0 %–38.6 %) in the low-risk group (score ≤ 4), 45.6 % (31/68; 95 % CI 34.0 %–57.4 %) in the intermediate-risk group ( >4 to <8 ), and 87.9 % (58/66; 95 % CI 78.0 %–94.1 %) in the high-risk group ( ≥8 ) ( p<0.001 ) (Fig. 3).

**Figure 3:**
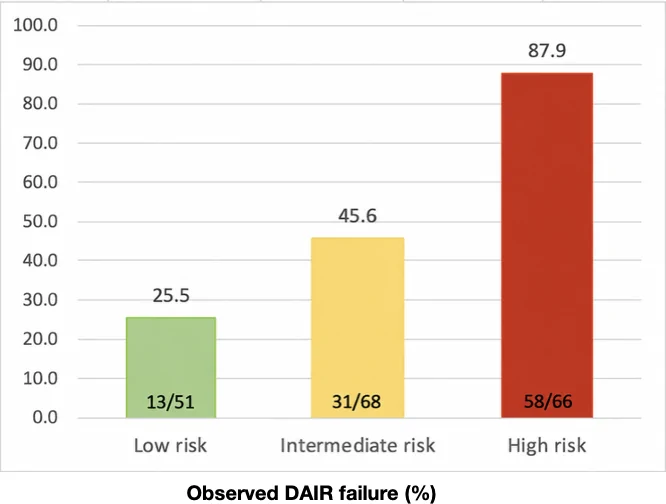
Observed DAIR failure across derived clinical score categories. Failure rates were 25.5 % (13/51; 95 % CI 15.0 %–38.6 %) in the low-risk group (score ≤ 4), 45.6 % (31/68; 95 % CI 34.0 %–57.4 %) in the intermediate-risk group ( >4 to <8 ), and 87.9 % (58/66; 95 % CI 78.0 %–94.1 %) in the high-risk group ( ≥8 ) ( p<0.001 ). Thresholds were empirically derived in the development cohort.

### 3.7 Sensitivity analyses

Sensitivity analyses supported the primary findings. Multiple imputation of missing predictor data yielded estimates of similar direction and magnitude, with all five predictors remaining statistically significant (Table S2). Failure rates were similar in 2006–2014 and 2015–2023 (53.3 % vs 57.5 %; p=0.63 ). Adjustment for calendar period showed no association between recruitment era and failure (2015–2023 vs 2006–2014: OR 1.33, 95 % CI 0.52–3.39; p=0.549 ) and did not materially alter the remaining predictor estimates (Table S3). When Gram-negative bacilli and polymicrobial infection were entered separately, both associations remained in the same direction (OR 2.58, 95 % CI 1.07–6.20 and OR 2.91, 95 % CI 0.85–9.92, respectively) (Table S4).

Excluding microbiological aetiology reduced discrimination to an AUC-ROC of 0.81 (95 % CI 0.74–0.87) for the multivariable model and 0.77 (95 % CI 0.71–0.84) for the corresponding reduced clinical score (Fig. S1 in the Supplement).

## 4 Discussion

In this multicentre cohort of patients with PJI treated with DAIR, the KLIC score did not demonstrate discriminative ability beyond chance, in line with previous external validation studies (Bernaus et al., 2022; Liukkonen et al., 2024; Löwik et al., 2018). In contrast, the newly developed model showed better discrimination in this cohort, with an AUC-ROC of 0.85 and satisfactory calibration. Internal validation suggested limited optimism, with an optimism-corrected AUC-ROC of 0.83 and a calibration slope of 0.91. The corresponding clinical score retained good discrimination (AUC-ROC 0.81) and identified groups with markedly different observed failure rates. These findings demonstrate prognostic information within this cohort but do not establish clinical utility, which requires external validation.

The lack of discrimination of the KLIC score in our cohort should not be interpreted as invalidating the original model but rather as highlighting limitations in its generalizability across different populations and outcome settings. KLIC was derived from 222 early postoperative PJIs collected between 1999 and 2014, and was designed to predict failure within 60 d after DAIR, with an observed failure rate of 23.4 % (Tornero et al., 2015). By contrast, our patients were followed for a minimum of 12 months after DAIR, with a median follow-up of 21.6 months, and the overall failure rate was 54 %. This longer outcome horizon allows the capture of relapses occurring after completion of antimicrobial therapy and may partly explain the difference in performance. Consistently, Liukkonen et al. (2024) reported similarly non-discriminatory performance of KLIC at 1 year (AUC 0.53).

Differences in the case mix may also contribute. Revision arthroplasty was more frequent in our cohort than in the KLIC derivation cohort (31.7 % vs 18.9 %), whereas coagulase-negative staphylococci (18.8 % vs 42.8 %) and polymicrobial infections (11.2 % vs 38.3 %) were less frequent (Tornero et al., 2015). Conversely, the proportion of cemented prostheses was almost identical (74.7 % vs 74.3 %), making cementation an unlikely explanation for the observed difference in performance. In addition, surgical and antimicrobial management of acute PJI has evolved since the period in which KLIC was developed. Taken together, these differences suggest that the performance of KLIC may depend on the similarity between the population and outcome horizon in which it is applied and those of its original derivation cohort.

The relatively high overall failure rate in our cohort should also be interpreted in this context. In addition to the minimum 12-month follow-up, our composite outcome classified the need for long-term suppressive antimicrobial therapy as failure. The multicentre design also incorporated a heterogeneous case mix. Differences in outcome definitions, duration of follow-up, patient selection, and centre-specific characteristics should therefore be considered when comparing DAIR success rates across studies.

The variables retained in the new model should be interpreted primarily as prognostic markers rather than causal determinants of DAIR failure. Their value lies in contributing information on the baseline probability of failure when DAIR is being considered; the present study cannot establish that modifying any individual predictor would alter the outcome. In particular, longer time from index arthroplasty to DAIR was associated with failure, consistent with previous studies (Davis et al., 2022; Espíndola et al., 2022), but this association should not be interpreted as evidence of a causal effect of treatment delay. This variable may also capture differences in clinical presentation, diagnostic complexity, referral pathways, or other influencing factors when DAIR is performed. Earlier intervention would therefore represent an actionable factor only when the delay itself is modifiable.

Comorbidity and CRP should be interpreted similarly. Although median CCI was low and identical in patients with and without failure, the association was concentrated among patients with CCI ≥ 3. This subgroup was small (23 patients overall and 19 in the complete-case model), resulting in a wide confidence interval around the effect estimate. Moreover, the ≥3 threshold was selected during model development after evaluating alternative parameterizations and therefore requires independent confirmation. CRP > 150 mg L^−1^ likewise provided prognostic information, and this threshold is supported by previous reports of DAIR outcomes (Wouthuyzen-Bakker et al., 2019); however, because alternative cut-points were explored in the present cohort, its predictive performance also requires external validation. These variables should be regarded as markers of baseline risk rather than as direct treatment targets.

Microbiological aetiology provided additional predictive information. Gram-negative bacilli and polymicrobial infections were combined in the primary model because both subgroups were relatively small and had similar observed failure rates. When analysed separately, their associations remained in the same direction and of comparable magnitude, arguing against the combined effect being driven solely by one category. Importantly, excluding microbiological aetiology reduced discrimination from 0.85 to 0.81 for the multivariable model and from 0.81 to 0.77 for the corresponding clinical score. Thus, microbiological information improves risk estimation when available before DAIR, although the remaining clinical variables retain predictive information when microbiological results are not yet available. These associations should again be interpreted prognostically rather than as evidence that a particular microorganism causally determines treatment failure.

This study has several strengths. It included patients from eight centres, used a mixed-effects modelling approach to account for centre-level clustering, assessed both discrimination and calibration, and incorporated bootstrap internal validation. Sensitivity analyses addressing missing data, calendar period, microbiological availability, and separation of Gram-negative and polymicrobial infections yielded results broadly consistent with the primary analysis. In particular, multiple imputation of missing predictor data produced estimates of similar direction and magnitude, and adjustment for calendar period did not materially alter the model.

Several limitations should nevertheless be acknowledged. First, the retrospective design may have introduced residual selection and information bias. Complete-case analysis excluded 39 patients, predominantly because of missing CRP values, although multiple-imputation sensitivity analysis supported the robustness of the primary estimates. Second, some predictor parameterizations, including CCI ≥ 3 and CRP > 150 mg L^−1^, as well as the score risk thresholds, were selected within the development cohort and may therefore be optimistic. Third, despite bootstrap internal validation, the model has not undergone external validation, and its calibration and discrimination may differ in other populations. The study was not designed to assess differential model performance across sociodemographic groups; potential differences in model fairness should therefore be examined during external validation. Fourth, the long recruitment period encompassed changes in PJI diagnosis and treatment, although failure rates and model estimates were not materially affected by adjustment for calendar period. Finally, the cohort included only patients who underwent DAIR. The model therefore estimates the risk of failure conditional on DAIR being performed; it cannot determine whether patients classified as high risk would have better outcomes with prosthesis exchange or another surgical strategy, nor does it account for intraoperative or postoperative factors, including surgical quality and antimicrobial management, that may influence the outcome.

If externally validated, the model could complement clinical judgement by providing an estimate of baseline failure risk when DAIR is being considered. However, the observed risk strata should not currently be interpreted as treatment-selection thresholds, and the present data do not establish that alternative surgery would improve outcomes in patients classified as high risk. External validation in independent PJI-DAIR cohorts is required to assess reproducibility, calibration, and clinical utility before implementation can be recommended.

## 5 Conclusions

In this multicentre cohort of patients with PJI treated with DAIR, the KLIC score did not demonstrate discriminative ability beyond chance. A newly developed model based on variables available before DAIR showed good discrimination and calibration, with limited optimism on internal validation. The corresponding clinical score identified groups with different observed risks of failure. External validation in independent PJI-DAIR cohorts is required before clinical implementation can be recommended.

## Supplement

10.5194/jbji-11-591-2026-supplement The supplement related to this article is available online at https://doi.org/10.5194/jbji-11-591-2026-supplement.

## Data Availability

The data underlying the findings of this study are available from the corresponding author upon reasonable request, subject to applicable data-protection regulations and institutional approval. The analytical code is available from the corresponding author upon reasonable request.

## References

[bib1.bib1] Abbaszadeh A, Yilmaz MK, Izadi N, Hoveidaei AH, Taheriazam A, Abedi AA, Parvizi J (2025). Efficacy of Debridement, Antibiotics, and Implant Retention in Total Hip and Knee Arthroplasty: A Systematic Review and Meta-Analysis. J Arthroplasty.

[bib1.bib2] Bernaus M, Auñón-Rubio Á, Monfort-Mira M, Arteagoitia-Colino I, Martínez-Ros J, Castellanos J, Lamo-Espinosa JM, Argüelles F, Veloso M, Gómez García L, Crespo FA, Sánchez-Fernández J, Murias-Álvarez J, Martí-Garín D, Hernández-González N, Villarejo-Fernández B, Valero-Cifuentes G, Hernández-Torres A, Molina-González J, Coifman-Lucena I, Esteban-Moreno J, Demaria P, Esteve-Palau E, del Pozo JL, Suárez Á, Carmona-Torre F, Darás Á, Baeza J, Font-Vizcarra L (2022). Risk Factors of DAIR Failure and Validation of the KLIC Score: A Multicenter Study of Four Hundred Fifty-Five Patients. Surg Infect (Larchmt).

[bib1.bib3] Collins GS, Moons KGM, Dhiman P, Riley RD, Beam AL, Van Calster B, Ghassemi M, Liu X, Reitsma JB, van Smeden M, Boulesteix AL, Camaradou JC, Celi LA, Denaxas S, Denniston AK, Glocker B, Golub RM, Harvey H, Heinze G, Hoffman MM, Kengne AP, Lam E, Lee N, Loder EW, Maier-Hein L, Mateen BA, McCradden MD, Oakden-Rayner L, Ordish J, Parnell R, Rose S, Singh K, Wynants L, Logullo P (2024). TRIPOD+AI statement: updated guidance for reporting clinical prediction models that use regression or machine learning methods. BMJ.

[bib1.bib4] Davis JS, Metcalf S, Clark B, Robinson JO, Huggan P, Luey C, McBride S, Aboltins C, Nelson R, Campbell D, Solomon LB, Schneider K, Loewenthal MR, Yates P, Athan E, Cooper D, Rad B, Allworth T, Reid A, Read K, Leung P, Sud A, Nagendra V, Chean R, Lemoh C, Mutalima N, Tran T, Grimwade K, Sehu M, Looke D, Torda A, Aung T, Graves S, Paterson DL, Manning L (2022). Predictors of Treatment Success After Periprosthetic Joint Infection: 24-Month Follow up From a Multicenter Prospective Observational Cohort Study of 653 Patients. Open Forum Infect Dis.

[bib1.bib5] Espíndola R, Vella V, Benito N, Mur I, Tedeschi S, Zamparini E, Hendriks JGE, Sorlí L, Murillo O, Soldevila L, Scarborough M, Scarborough C, Kluytmans J, Ferrari MC, Pletz MW, Mcnamara I, Escudero-Sanchez R, Arvieux C, Batailler C, Dauchy FA, Liu WY, Lora-Tamayo J, Praena J, Ustianowski A, Cinconze E, Pellegrini M, Bagnoli F, Rodríguez-Baño J, del Toro MD, Cuperus N, Manfré G, Suárez-Barrenechea AI, Pascual-Hernandez A, Rivera A, Crusi X, Jordán M, Rossi N, vande Kerkhof T, Horcajada JP, Gómez-Junyent J, Alier A, van Rijen M, Romme J, Ankert J, Whitehouse C, Jones A, Cobo J, Moreno J, Meheut A, Gledel C, Perreau P, van Wensen RJA, Lindergard G (2022). Rates and Predictors of Treatment Failure in Staphylococcus aureus Prosthetic Joint Infections According to Different Management Strategies: A Multinational Cohort Study – The ARTHR-IS Study Group. Infect Dis Ther.

[bib1.bib6] Jiménez-Garrido C, Gómez-Palomo JM, Rodriguez-Delourme I, Durán-Garrido FJ, Nuño-Álvarez E, Montañez-Heredia E (2018). The Kidney, Liver, Index surgery and C reactive protein score is a predictor of treatment response in acute prosthetic joint infection. Int Orthop.

[bib1.bib7] Kristensen NK, Callary SA, Nelson R, Harries D, Lorimer M, Smith P, Campbell D (2025). Outcomes of Debridement, Antibiotics, and Implant Retention in the Management of Infected Total Knee Arthroplasty: Analysis of 5, 178 Cases From the National Australian Registry. J Arthroplasty.

[bib1.bib8] Lenguerrand E, Whitehouse MR, Beswick AD, Kunutsor SK, Foguet P, Porter M, Blom AW (2019). Risk factors associated with revision for prosthetic joint infection following knee replacement: an observational cohort study from England and Wales. Lancet Infect Dis.

[bib1.bib9] Liukkonen R, Honkanen M, Eskelinen A, Reito A (2024). KLIC Score Does Not Predict Failure After Early Prosthetic Joint Infection: An External Validation With 153 Knees and 130 Hips. J Arthroplasty.

[bib1.bib10] Löwik CAM, Jutte PC, Tornero E, Ploegmakers JJW, Knobben BAS, de Vries AJ, Zijlstra WP, Dijkstra B, Soriano A, Wouthuyzen-Bakker M (2018). Predicting Failure in Early Acute Prosthetic Joint Infection Treated With Debridement, Antibiotics, and Implant Retention: External Validation of the KLIC Score. J Arthroplasty.

[bib1.bib11] Löwik CAM, Parvizi J, Jutte PC, Zijlstra WP, Knobben BAS, Xu C, Goswami K, Belden KA, Sousa R, Carvalho A, Martínez-Pastor JC, Soriano A, Wouthuyzen-Bakker M (2020). Debridement, Antibiotics, and Implant Retention Is a Viable Treatment Option for Early Periprosthetic Joint Infection Presenting More Than 4 Weeks After Index Arthroplasty. Clin Infect Dis.

[bib1.bib12] MAC – The McMaster Arthroplasty Collaborative (2020). Risk Factors for Periprosthetic Joint Infection Following Primary Total Hip Arthroplasty. J Bone Joint Surg.

[bib1.bib13] McNally M, Sousa R, Wouthuyzen-Bakker M, Chen AF, Soriano A, Vogely HC, Clauss M, Higuera CA, Trebše R (2021). The EBJIS definition of periprosthetic joint infection. Bone Joint J.

[bib1.bib14] Parel PM, Chiu AK, Zhao AY, Agarwal AR, Gu A, Marrache M, Thakkar SC, Golladay GJ (2025). Have We Succeeded in Reducing the 2-Year Periprosthetic Joint Infection Incidence Rate Following Total Hip Arthroplasty? A National Database Analysis from 2011 to 2019. J Arthroplasty.

[bib1.bib15] Patel R (2023). Periprosthetic Joint Infection. New Engl J Med.

[bib1.bib16] Quinlan ND, Werner BC, Brown TE, Browne JA (2020). Risk of Prosthetic Joint Infection Increases Following Early Aseptic Revision Surgery of Total Hip and Knee Arthroplasty. J Arthroplasty.

[bib1.bib17] Rahardja R, Zhu M, Davis JS, Manning L, Metcalf S, Young SW (2023). Success of Debridement, Antibiotics, and Implant Retention in Prosthetic Joint Infection Following Primary Total Knee Arthroplasty: Results From a Prospective Multicenter Study of 189 Cases. J Arthroplasty.

[bib1.bib18] Sigmund IK, Wouthuyzen-Bakker M, Ferry T, Metsemakers WJ, Clauss M, Soriano A, Trebse R, Sousa R (2025). Debridement, antimicrobial therapy, and implant retention (DAIR) as curative surgical strategy for acute periprosthetic hip and knee infections: a summary of the position paper from the European Bone & Joint Infection Society (EBJIS). J Bone Joint Infect.

[bib1.bib19] Tornero E, Morata L, Martínez-Pastor JC, Bori G, Climent C, García-Velez DM, García-Ramiro S, Bosch J, Mensa J, Soriano A (2015). KLIC-score for predicting early failure in prosthetic joint infections treated with debridement, implant retention and antibiotics. Clin Microbiol Infect.

[bib1.bib20] Wouthuyzen-Bakker M, Sebillotte M, Lomas J, Taylor A, Palomares EB, Murillo O, Parvizi J, Shohat N, Reinoso JC, Sánchez RE, Fernandez-Sampedro M, Senneville E, Huotari K, Barbero JM, Garcia-Cañete J, Lora-Tamayo J, Ferrari MC, Vaznaisiene D, Yusuf E, Aboltins C, Trebse R, Salles MJ, Benito N, Vila A, Toro MDDel, Kramer TS, Petersdorf S, Diaz-Brito V, Tufan ZK, Sanchez M, Arvieux C, Soriano A (2019). Clinical outcome and risk factors for failure in late acute prosthetic joint infections treated with debridement and implant retention. J Infect.

